# New Assessment Tool—Postpartum Functional Assessment Questionnaire

**DOI:** 10.3390/medicina59071219

**Published:** 2023-06-29

**Authors:** Manuela Filipec, Vladimir Blagaić, Marinela Jadanec Đurin, Paulo Zekan, Pero Hrabač, Ana Zovko

**Affiliations:** 1 Department of Physiotherapy, University North, 42000 Varaždin, Croatia; 2 Department of Obstetrics and Gynecology, Clinic for Gynecology and Obstetrics, School of Medicine, University of Zagreb, Clinical Hospital “Sveti Duh”, 10000 Zagreb, Croatia; vladimir.blagaic@gmail.com; 3 Department of Physical Medicine and Rehabilitation, Clinical Hospital “Sveti Duh”, 10000 Zagreb, Croatia; marinela.jadanec@gmail.com; 4 Clinic for Gynecology and Obstetrics, Clinical Hospital “Sveti Duh”, 10000 Zagreb, Croatia; paulo.zekan@gmail.com (P.Z.); ana.zovko88@gmail.com (A.Z.); 5 Department of Medical Statistics, Epidemiology and Medical Informatics, “Andrija Štampar” School of Public Health “Andrija Štampar”, 10000 Zagreb, Croatia; pero.hrabac@mef.hr; 6 School of Medicine, University of Zagreb, 10000 Zagreb, Croatia

**Keywords:** childbirth, functional abilities, pain intensity

## Abstract

*Background and Objectives*: Functional status of the mother after delivery is crucial for performing activities of daily living and caring for the newborn. It is important to assess functional abilities after childbirth in order to improve the quality of postpartum care. The aim of this study is to determine the psychometric properties of the questionnaire and assess the functional abilities after childbirth. *Materials and Methods*: This study is observational. Postpartum Functional Assessment Questionnaire includes eleven items. 301 women after childbirth, 234 after vaginal birth and 67 after caesarean section participated in the study. An assessment of pain intensity and functional abilities was performed on the first and third day after childbirth. The Factor and Cronbach’s alpha analyses were performed to determine the factor structure and internal consistency. *Results*: The analysis reveals two factors, with seven items loading on factor 1 and four on factor 2. Cronbach’s alpha for construct I (Mobility) at the first day was 0.927 and at the third day was 0.913; and for Factor II (Self-care) at the first day was 0.846 and at the third day was 0.894. All between-group differences in pain intensity and functional abilities were highly statistically significant (*p* < 0.001). Differences between the first and third postpartum day were statistically significant for all variables and all subgroups (*p* < 0.001). *Conclusions*: Postpartum Functional Assessment Questionnaire has good psychometric properties and is a valuable tool for use in clinical practice.

## 1. Introduction

Functional status has an impact on health and wellbeing [1] It refers to the ability to perform daily life activities and after childbirth it includes the additional domain, of the infant care. As such, functional status after childbirth is defined as the ability of the mother to care for the child, as well as the ability to care for herself, the household, social, and professional activities [1]. The mode of delivery is thought to be related to the time required for recovery, which may contribute to functional limitations after childbirth [2,3]. Recovery after caesarean section is associated with higher pain intensity and slower recovery compared to recovery after vaginal delivery due to abdominal wall disruption [4]. Also, after a caesarean section and vaginal delivery, muscle strength is reduced, trunk stability is impaired and fatigue is present, which affects functional status [5]. The mode of delivery due to individual and sociomedical factors affects outcomes and functional status after childbirth [6].

Childbirth causes difficulties and disability in performing daily activities in postpartum period. The appearance of pain after childbirth directly affects the functional abilities of the mother (sitting, getting up, walking, personal care, etc.) and ability to care for the child (changing diapers, breastfeeding, etc.). The ability of optimal function during the postpartum period is vital and is an indicator of successful recovery after delivery. 

The aim of this study was to determine the psychometric properties of the questionnaire and assess the functional abilities after vaginal delivery and caesarean section.

## 2. Materials and Methods

Observational trial was conducted to assess the functional abilities after childbirth in Clinic for Gynaecology and Obstetrics, Clinical hospital “Sveti Duh” in Zagreb, Croatia from January to April 2021. The study was approved by Ethical Committee of the hospital “Sveti Duh”. All women were offered to participate in the study after childbirth. Those who agree to take part had to sign informed consent and to fill out a questionnaire. Exclusion criteria were rheumatic diseases, neurological diseases and any other disease whose symptoms can cause disability.

### 2.1. Participants

Primiparous and multiparous, singleton and multiple pregnancy, ages between 18 and 45 years. Participants were divided into four study groups according to the mode of birth: vaginal birth without any trauma (V), vaginal birth with 1° or 2° perineal rupture (R), vaginal birth with episiotomy (E) and caesarean section (C). The patients received analgesic therapy as needed (non-steroidal anti-rheumatic drugs—NSAIDs and paracetamol). In the study, 127 patients had vaginal delivery with epidural analgesia, 7 caesarean sections under general anesthesia, 58 caesarean sections under spinal anesthesia and 2 cesarean sections under epidural anesthesia. The epidural catheter was removed 2 h after delivery. There were two operative deliveries (vacuum extractions) in the study, but they were excluded due to the incompletely filled questionnaire.

### 2.2. Questionnaire

The Postpartum Functional Assessment Questionnaire, designed specifically for this study, was used to assess functional abilities. 

The questionnaire consists of demographic data (year of birth, body weight, height, body mass index (BMI), education level), gynaecological history (pregnancy and childbirth data), pain intensity assessment (Numeric Pain Rating Scale), and functional ability assessment (Postpartum Functional Assessment Questionnaire). 

The pain intensity was assessed by Numeric pain rating scale. The scale assesses the severity of pain on a numerical scale from 0 to10, where 0 means no pain, 1–3 means mild pain, 4–6 means moderate pain and 7–10 means severe pain. 

Postpartum Functional Assessment Questionnaire includes assessment of functional abilities related to mobility (turning to the side [variable 1; v1], sitting [v2], getting up [v3], walking [v2]), personal care (self-care/body care/showering) [v5], fluid intake [v6], food intake [v7], sleep [v8], oral care/brushing teeth [v9], going to the toilet [v10]) and child care (lifting / carrying a child) [v11]). Abilities were assessed on a scale from 0 to 3 where 0 indicates the performance of activities without difficulty, 1 indicates the performance of activities with minimal difficulty, 2 indicates the performance of activities of moderate difficulty, 3 indicates the inability to perform independently activities/necessary help. Each construct of functional ability was assessed separately by summing scores. 

Assessment of pain intensity and functional abilities was performed on first and third postpartum day. Pain intensity and functional abilities assessment was done in order to compare the course of the postpartum recovery. 

### 2.3. Statistical Analysis

R program (version 4.1.3, The R Foundation for Statistical Computing, Vienna, Austria) was used for data analysis. Sample size was obtained by power analysis with estimated size of d = 0.05 significance level α = 0.05 and power of 80%. The reliability and validity of the questionnaire was examined through Cronbach’s alpha, primary factor analysis and Kaiser-Meyer-Olkin measure of sampling adequacy. The Kolmogorov-Smirnov test was used to test normality of distribution. 

Data were expressed as mean with 95% confidence interval and proportions for numerical and categorical variables respectively. Difference between groups in Numeric pain rating scale score and Functional abilities score were analysed by one-way ANOVA test with post-hoc t-test. Impact of age, parity, birth mode, body mass index and education level on Numeric pain rating scale and Functional abilities score were analysed using multivariate linear regression. Categorical variables included in linear regression were dummy coded. *p*-value of less than 0.05 was considered statistically significant.

## 3. Results

**Figure 1:**
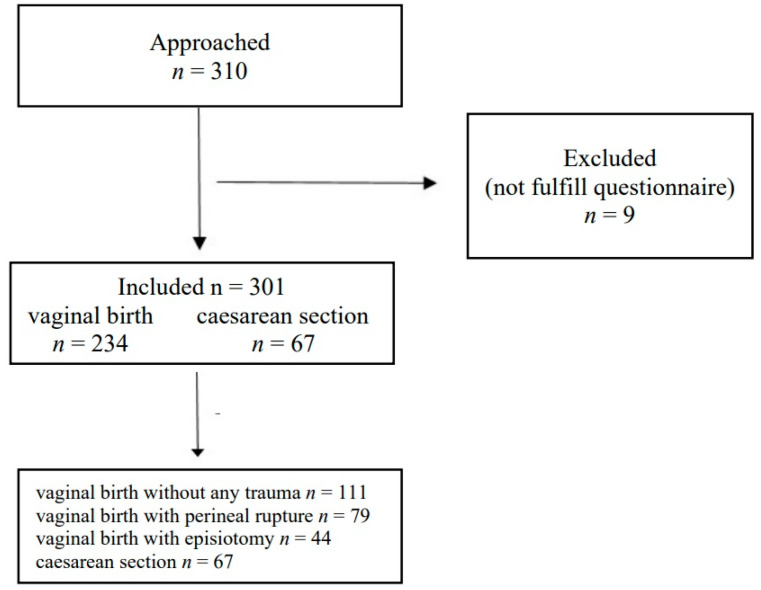
Flow of participants.

The flow of participants through the study is presented at Figure 1. Among 310 approached participants from January to April 2021, 301 participants were included in the study. Other 9 were excluded because did not fulfill the entire questionnaire.

### 3.1. Factor Analysis

Principal component analysis (PCA) technique was used to explore the validity of each construct and of the questionnaire as a whole. Sampling adequacy was tested by Kaiser-Meyer-Olkin (KMO) measure. KMO measure for variables at the first day was 0.921 and at third postpartum day was 0.907 with Bartlett’s sphericity test value of <0.001 on both days.

Table 1 show a rotated component matrix (Varimax orthogonal rotation) for two components identified by the PCA with load coefficients above 0.5 bolded. After the PCA, it was obvious that the questionnaire assesses two factors. The Factor I is called Mobility (variables 1, 2, 3, 4, 5, 10 and 11) and the Factor II is called Self-care (variables 6, 7, 8 and 9). These two factors explain 67.9% of total variance. 

Rotated component matrix shows situation at day 3 to be comparable with the one seen on day 1, with the two factors explaining 67.3% of the total variance. 

### 3.2. Internal Consistency

The internal consistency of Factor I (7 items) or Mobility at the first day was 0.927 and at the third day was 0.913 and for Factor II (Self-care) at the first day was 0.846 and at the third day was 0.894.

### 3.3. Clinical Outcome 

Demographic and obstetric characteristics are shown in Table 2. 

Numeric pain rating scale scores and functional abilities are shown in Table 3. All between-group differences were highly statistically significant (*p* < 0.001).

Post-hoc analysis revealed that the statistical significance arises from the following differences: C > V; C > R; E > V and E > R (Table 4). This was true for all variables in Table 4 except for self-care at the third day where the only differences were C > V and C > R. Differences between the first and third day were statistically significant for all variables and all subgroups (*p* < 0.001). In the study, there were 40 elective caesarean sections and 27 secondary caesarean sections and there is no difference in functional abilities between them.

Relationship of birth mode, age, education level, body mass index and parity with pain intensity and functional abilities are shown in Table 5.

A linear multiple regression was performed to explore the effect of predictor variables on the outcomes. As the predictor variables we used age (years), BMI (kg/m^2^), parity (number of previous births), education level (dummy coded as 0 for elementary school, 1 for high school and 2 for faculty and above) and birth mode (dummy coded as 0 for vaginal birth without trauma, 1 for vaginal birth with perineal rupture, 2 for vaginal birth with episiotomy and 3 for caesarean section).

Outcome (dependent) variables were pain intensity, mobility and self-care at the first and third day. Linear regression using all five mentioned predictors was able to account for some variance changes in all six variables with adjusted R^2^ values ranging from 0.168 for pain intensity at the third day to 0.274 for mobility at the third day. The exception was self-care at the third day with adjusted R^2^ value of 0.042. Using more advanced regression methods (forward and backward stepwise), the obtained adjusted R^2^ values were: 0.203 for pain intensity at the first day; 0.173 for pain intensity at the third day; 0.259 for mobility at the first day; 0.202 for mobility at the third day; 0.196 for self-care at the first day and 0.034 for self-care at the third day. In all of the six models mentioned in the previous sentence, only birth mode was selected by the stepwise method as a relevant independent variable. 

## 4. Discussion

This study demonstrates that Postpartum Functional Assessment Questionnaire provides satisfactory psychometric properties and can be used to assess functional abilities after childbirth.

The female body changes during the early postpartum period returning the different body systems to the pre-pregnancy state [2]. Eisenach suggest that approximately 10% of women have persistent pain after delivery, which interfered with their activities of everyday life [7].

Pain and physical activity are tightly intertwined. Activity and function are basic in the assessment of functioning after childbirth. In the literature there is a scarcity of data about assessment of functional status in the early postpartum period after vaginal delivery and caesarean section [8].

Our results clearly demonstrate association between birth mode and pain intensity and degree of functional disability in early postpartum period. Analysis by groups revealed that vaginal birth without any trauma and vaginal birth with low grade perineal rupture had same effect on all outcomes in this research. Although, perineal rupture is small wound requiring a few sutures it still created significant discomfort on first postpartum day when compared to no rupture. Our findings are in correlation with other studies which indicates pain after delivery which is causing functional limitations in everyday activities of mother [2,8].

Pereira indicates that birth mode is linked to a woman’s recovery time and thus may contribute to functional limitations in everyday activities. Vaginal delivery may result in trauma and perineal discomfort [2] Labor and delivery are processes which are associated with microscopic or gross tissue injury to the mother [7] which can lead to limitations of mother in the activities of daily living. 

Results of our study indicates that episiotomy produced equal level of pain intensity and functional disability as caesarean section. An episiotomy has been described as a triggering factor for complication, such as perineal laceration and pain in perineal region. Pereira suggest that pain after perineal trauma restricted women when perform everyday activities such as sitting down, walking, sleeping and caring for a newborn [2]. Comparable pain intensities may be surprising since episiotomy wound is smaller and operating time is shorter when compared to caesarean section. However, this may warrant obstetricians and midwifes not to perform episiotomy routinely and to carefully weigh benefits and harms related to episiotomy. In addition, this finding may alert attending physicians and nurses at maternity wards about sufficient doses of analgesics in women with episiotomy. 

Pereira et al., indicate that compared with woman who had unassisted vaginal deliveries, the risk of a reduction in health and well-being in the early postpartum period is higher among women who have had some kind of unplanned care, such as the use of forceps during delivery [2]. While Scharpe point to the connection between pain and activity, ie functioning in the activities of everyday life, is strongly connected after childbirth by caesarean section [8]. This fact points to the great impact that caesarean section as an operation leaves on the pain and functioning of the mother [8]. Pain present in caesarean postpartum women is related to several factors such as surgical incision, which involves several layers of tissue that need to go through repair process, contractions of uterine involution process and cramping stimulated by breastfeeding, presence of gases and intestinal constipation. So, it is not surprising that pain occurs in mothers who have given birth by caesarean section. 

Presence of pain during the puerperium makes it difficult for women to perform the daily activities required during this time period, such as self-care, newborn care, mobility which can be reflected in physical, psychological and emotional issues. Sharpe suggests a relatively long period of time it takes a mother to return to pre-pregnancy levels of functioning [8]. This points to the importance of assessing the function of the mothers in the activities of everyday life and to provide maximum care so that they can return as early as possible in everyday activities [8].

Multivariate linear regression analysis did not found age, body mass index and education level to be associated with postpartum pain and functional disability. Although the relationship between parity and almost all outcomes was found statistically significant in analysis, when the results were adjusted for birth mode the relationship was statistically significant. This is not surprising since it is known that birth mode is associated with parity. Furthermore, we believe that multiparous woman used their experience from previous pregnancies to accommodate better at maternity ward which produced lower level of functional disability at first postpartum day. 

Our results are consistent with the results of Pereira et al. [2]. Also, Pereira et al. point that the highest number of complaints was associated with movement activities and caesarean section postpartum but they didn’t find relationship between functional limitations and parity in their study [2]. Rowlands et al., think that birth mode is associated with differences in outcomes at three months postpartum [6]. By comparison to women who had unassisted vaginal births, the risk of reduced postnatal health and wellbeing was higher among the women who had forceps-assisted vaginal births [6].

Women should receive special attention during early postpartum period because pain after delivery interfere with functional daily activities during puerperium, and therefore, with their quality of life [2]. The pain experienced in the postpartum period restrict the daily activities related to mobility, self-care and newborn care^7^ which is why it is extremely important to consider the mother’s ability to perform activities of daily living and possible limitations. The emphasis is on early assessment with the aim of preventing limitations in the mother’s quality of life. 

As far as we know this is the first time that different modalities of vaginal birth (without rupture, with low degree perineal rupture and episiotomy) are compared. In addition, the results are in accordance with previous study reporting greater pain and functional disability after caesarean section when compared to vaginal birth [2].

The limitation of the study is relatively short follow-up interval (only three days) so we were unable to examine how birth modality influenced pain intensity and functional disability throughout whole puerperium.

The value of this study is in the development and clinical application of tool for assessing functional abilities after childbirth, which contributes to the quality of postpartum care.

## 5. Conclusions

The results of this study indicate that Postpartum Functional Assessment Questionnaire seems to be a valid and necessary tool in assessing the functional abilities after childbirth.

## Figures and Tables

**Table 1:** Rotated component matrix.

	Rotated Component Matrix at Day 1		Rotated Component Matrix at Day 3	
Factor	1	2	1	2
v3_standing	0.881	0.159	0.884	0.097
v4_walking	0.850	0.279	0.807	0.265
v2_sitting	0.802	0.114	0.783	0.178
v5_peronal care	0.749	0.363	0.714	0.289
v1_turning to the side	0.745	0.319	0.814	0.275
v13_lifting	0.703	0.466	0.701	0.412
v10_toilet	0.670	0.464	0.579	0.447
v7_food intake	0.173	0.895	0.249	0.910
v6_liquid intake	0.158	0.889	0.234	0.913
v9_oral care	0.375	0.771	0.180	0.901
v8_sleep	0.227	0.559	0.409	0.587
v11_child care	0.428	0.514	0.493	0.384

**Table 2:** Demographic and obstetric characteristics of participants.

	*N* = 301 (100%)
Birth mode	Vaginal birth without any trauma *n* = 111 (37%)
	Vaginal birth with perineal rupture *n* = 79 (26%)
	Vaginal birth with episiotomy *n* = 44 (15%)
	Caesarean section *n* = 67 (22%)
Parity	Primiparous *n* = 134 (45%)
	Multiparous *n* = 167 (55%)
Age	33.4 (32.9–33.9) years
Body mass index	28.4 (27.9–28.9)
Education level	Completed high school *n* = 87 (29%)
	Completed 3-year university programme *n* = 32 (11%)
	Completed 5-year university programme *n* = 182 (60%)

Values are given as number (percentage).

**Table 3:** Numeric pain rating scores and functional abilities scores for all groups.

	Vaginal Birth without any Trauma	Vaginal Birth with Perineal Rupture (1° and 2°)	Vaginal Birth with Episiotomy	Caesarean Section	*p*-Value
Pain Intensity at Day 1	3.65 (3.26–4.06)	4.58 (4.06–5.10)	5.59 (4.92–6.26)	6.49 (5.97–7.01)	<0.001
Pain Intensity at Day 3	1.32 (1.02–1.62)	1.67 (1.31–2.04)	2.89 (2.25–3.52)	3.19 (2.81–3.58)	<0.001
Mobility at Day 1	4.31 (3.76–4.86)	5.41 (4.81–6.00)	7.73 (7.27–8.18)	7.91 (7.21–8.61)	<0.001
Mobility at Day 3	1.75 (1.34–2.16)	2.19 (1.69–2.69)	4.80 (3.94–5.65)	4.30 (3.66–4.94)	<0.001
Self-Care at Day 1	2.54 (2.06–3.02)	3.28 (2.62–3.93)	5.41 (4.40–6.42)	7.52 (6.33–8.71)	<0.001
Self-Care at Day 3	0.80 (0.44–1.16)	0.97 (0.49–1.46)	2.48 (1.51–3.45)	2.91 (2.07–3.75)	<0.001
Child-Care at Day 1	2.26 (1.85–2.67)	2.66 (2.14–3.18)	4.50 (3.75–5.25)	5.70 (4.98–6.42)	<0.001
Child-Care at Day 3	1.16 (0.86–1.46)	1.13 (0.79–1.46)	1.89 (1.23–2.54)	2.88 (2.26–3.50)	<0.001

Data are given as mean with 95%. *N*= 301. *p*-value was obtained by one-way ANOVA test.

**Table 4:** Results of post-hoc t-test analysis for all outcomes.

	V vs. R	V vs. E	V vs. C	R vs. E	R vs. C	E vs. C
Pain Intensity at Day 1	0.03	<0.001	<0.001	0.09	<0.001	0.21
Pain Intensity at Day 3	0.96	<0.001	<0.001	<0.001	<0.001	1.00
Mobility at Day 1	0.04	<0.001	<0.001	<0.001	<0.001	1.00
Mobility at Day 3	1.00	<0.001	<0.001	<0.001	<0.001	1.00
Self-Care at Day 1	0.84	<0.001	<0.001	0.006	<0.001	0.009
Self-Care at Day 3	1.00	0.002	<0.001	0.01	<0.001	1.00
Child-Care at Day 1	1.00	<0.001	<0.001	<0.001	<0.001	0.07
Child-Care at Day 3	1.00	0.20	<0.001	0.21	<0.001	0.04

Groups: V—Vaginal birth without any trauma, R—Vaginal birth with perineal rupture, E—Vaginal birth with episiotomy, C—C-section.

**Table 5:** Results for relationship of birth mode, age, education level, body mass index and parity with Numeric pain rating scores and Functional abilities scores.

Variable	Numeric Pain Rating Scale Day 1		Numeric Pain Rating Scale Day 3		Mobility Day 1		Mobility Day 3	
	Parameter Estimate with 95% CI	*p*-Value	Parameter Estimate with 95% CI	*p*-Value	Parameter estimate with 95% CI	*p*-Value	Parameter Estimate with 95% CI	*p*-Value
Birth mode	0.95 (0.74 to 1.16)	<0.05 *	0.66 (0.50 to 0.82)	<0.05 *	1.29 (1.03 to 1.55)	<0.05 *	0.98 (0.74 to 1.22)	<0.05 *
Age	−0.02 (−0.07 to 0.04)	0.59	0.00 (−0.04 to 0.04)	0.87	−0.19 (−0.09 to 0.05)	0.60	0.00 (−0.06 to 0.06)	0.90
Education level	0.27 (−0.04 to 0.58)	0.09	0.21 (−0.02 to 0.45)	0.07	0.51 (0.12 to 0.90)	<0.05	0.31 (−0.03 to 0.65)	0.08
Body mass index	0.03 (−0.04 to 0.09)	0.42	0.03 (−0.02 to 0.08)	0.25	0.00 (−0.08 to 0.08)	0.98	0.03 (−0.04 to 0.10)	0.42
Parity	−0.48 (−075 to −0.20)	<0.05	−0.32 (−0.53 to −0.11)	<0.05	−0.94 (−1.27 to −0.60)	<0.05 *	−0.52 (−0.82 to −0.21)	<0.05
**Variable**	**Self-Care Day 1**		**Self-Care Day 3**		**Child-Care Day 1**		**Child-Care Day 3**	
	**Parameter Estimate with 95% CI**	***p*** **-Value**	**Parameter Estimate with 95% CI**	***p*** **-Value**	**Parameter Estimate with 95% CI**	***p*** **-Value**	**Parameter Estimate with 95% CI**	***p*** **-Value**
Birth mode	1.66 (1–33 to 1.99)	<0.05 *	0.75 (0.50 to 1.00)	<0.05	1.17 (0.94 to 1.41)	<0.05 *	0.56 (0.38 to 0.75)	<0.05 *
Age	0.00 (−0.09 to 0.09)	0.92	0.25 (−0.04 to 0.09)	0.45	0.01 (−0.06 to 0.07)	0.75	0.04 (−0.01 to 0.09)	0.12
Education level	0.21 (−0.29 to 0.71)	0.41	−0.19 (−0.54 to 0.16)	0.29	0.46 (0.11 to 0.82)	<0.05	0.19 (−0.07 to 0.44)	0.16
Body mass index	0.09 (−0.01 to 0.19)	0.07	0.05 (−0.02 to 0.12)	0.14	0.04 (−0.03 to 0.11)	0.28	0.02 (−0.03 to 0.07)	0.44
Parity	−0.78 (−1.22 to −0.35)	<0.05	−0.24 (−0.55 to 0.07)	0.13	−0.81 (−1.12 to −0.50)	<0.05 *	−0.35 (−0.57 to −0.12)	<0.05

* Statistically significant relationship from multivariate linear regression analysis.

## Data Availability

Not applicable.
